# Case Report: a rare primary gastric choriocarcinoma revealed on ^18^F-FDG PET/CT

**DOI:** 10.3389/fonc.2023.1227236

**Published:** 2023-11-09

**Authors:** Yi Zhao, Wei Diao, Suping Li, Mengxi Yang, Zhuzhong Cheng

**Affiliations:** ^1^ Department of Nuclear Medicine, Affiliated Hospital of North Sichuan Medical College, Nanchong, Sichuan, China; ^2^ Department of Nuclear Medicine, Sichuan Clinical Research Center for Cancer, Sichuan Cancer Hospital & Institute, Sichuan Cancer Center, Affiliated Cancer Hospital of University of Electronic Science and Technology of China, Chengdu, Sichuan, China; ^3^ Department of Radiology, Sichuan Clinical Research Center for Cancer, Sichuan Cancer Hospital & Institute, Sichuan Cancer Center, Affiliated Cancer Hospital of University of Electronic Science and Technology of China, Chengdu, Sichuan, China

**Keywords:** primary gastric choriocarcinoma, gastric cancer, hepatic metastases, 18 F-FDG PET/CT, case report

## Abstract

Choriocarcinoma is an exceptionally aggressive trophoblastic cell tumor that that typically originates in gonadal tissues, with rare occurrences outside the gonads, including the mediastinum, retroperitoneum, and intracranial sites. However, it rarely occurs in the stomach. Herein, we presented a case of primary gastric choriocarcinoma in a 27-year-old female patient who found multiple liver masses detected during physical examination, accompanied by remarkably elevated human chorionic gonadotropin levels. The ^18^F-FDG PET/CT scan suggested ring-shaped intense uptake masses located in the gastric sinus and liver, and no significance in the pelvic region. Final histopathology indicated primary choriocarcinoma of the stomach. This case illustrates that ^18^F-FDG PET/CT is an essential imaging technique for the clinical diagnosis and stage of primary choriocarcinoma.

## Introduction

Choriocarcinoma is a highly malignant and aggressive tumor of the trophoblastic cells, including two subtypes: gestational choriocarcinoma and nongestational choriocarcinoma (1–3). Nongestational choriocarcinoma, also known as primary choriocarcinoma, is an extremely rare and highly malignant and aggressive tumor, with a very poor prognosis and a tendency to develop early distant organ metastasis (4). Owing to the rarity and nonspecific symptoms of primary choriocarcinoma, the clinical diagnosis is often difficult.

Primary choriocarcinoma mainly originates from germ cells in the gonads, and more rarely occurs outside the gonads which mainly locating in midline areas of the body, such as the lungs, mediastinum, retroperitoneum and pineal gland (5). Primary gastric choriocarcinoma (PGC) is an extremely rare aggressive tumor, that represents 0.08% of all gastric cancers (6). In this case, we describe a rare PGC case with multiple liver metastasis revealed in physical examination.

## Case description

A 27-year-old woman initially presented to local hospital with complains suggestive of COVID-19. During her evaluation, the chest computed tomography (CT) scan accidently detected the multiple liver masses. Subsequently, an abdomen CT scan was conducted, which raised suspicion that the liver masses may be metastatic from the gastric cancer. To explore this possibility further, a gastroscopy was performed, revealing a conspicuous, curved bulging neoplasm of the gastric sinus with central part depression and a well-defined ulcerated peripheral bulge. Corresponding biopsy results confirmed the low-differentiated carcinoma with histological and immunophenotypic characteristics of choriocarcinoma. Following these findings, the patient was referred to our hospital for further evaluation and treatment. We conducted a comprehensive array of diagnostic examinations, including hematological tests, tumor marker assessments, and measurement of human chorionic gonadotropin (β-HCG) levels. The hematology analysis primarily indicated abnormalities related to red blood cells, notably moderate anemia, which decreased for RBC, Hb (69 g/L), MCV, mMCH, MCHC, RDW, along with increased RET and IRF. Other hematology indicators such as leucocyte, neutrophils and lymphocyte, had no significant deviations from the norm. As for the tumor markers, only the NSE was increased, while other markers like CEA, AFP, ProGRP, CA125 remained within the normal range. Remarkably, the β-HCG was elevated over 10000 mIU/ml. Based on these findings, the patient received a preliminarily diagnosed as primary gastric choriocarcinoma (PGC). However, given the rarity of this disease, further investigations were necessary to differentiate between primary choriocarcinoma and metastatic disease. Consequently, magnetic resonance imaging (MRI) and positron emission tomography/computed tomography (PET/CT) were performed to facilitate a more comprehensive and accurate differential diagnosis.

**Figure 1:**
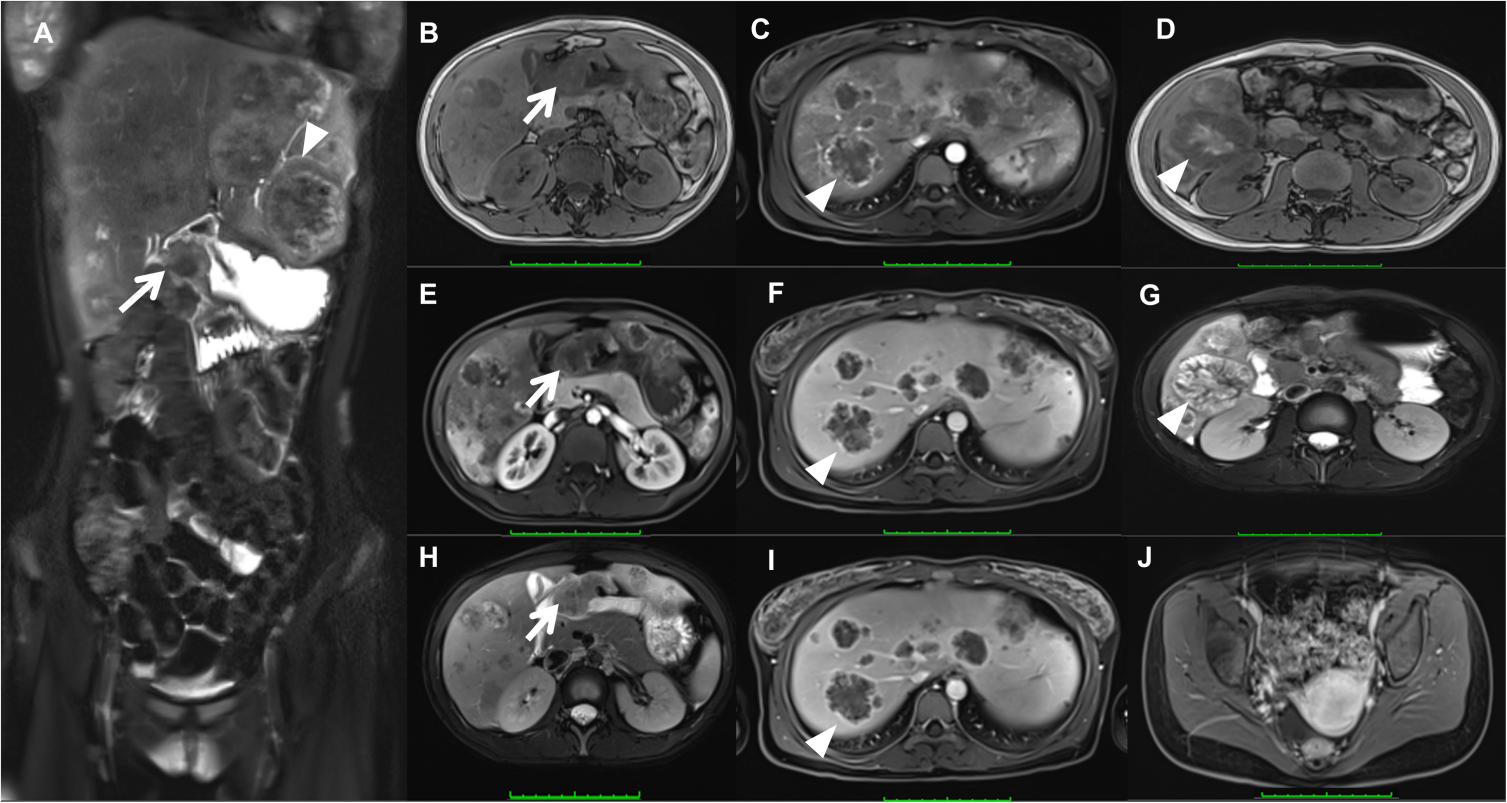
Magnetic resonance imaging (MRI) showed heterogeneous thickened of the gastric sinus wall (arrow) on T1WI **(B)**, T2WI **(A, H)**, and DEC-MRI **(E)**; Multiple intrahepatic masses (triangles) demonstrated on T1WI **(D)**, T2WI **(A, G)**, and DEC-MRI (arteria phase, **(C)**; portal venous phase, **(F)**; delayed phase, **(I)**. No apparent lesion was discovered in the uterus **(J)**.

As presented in **Figure 1**, the MRI scan showed heterogeneous thickened of the gastric sinus wall with the isointense or hypointense signal on T1WI (**Figure 1B**), the isointense or hyperintense signal on T2WI (**Figures 1A, H**), and heterogeneous and mild enhancement on dynamic contrast-enhanced MRI (DEC-MRI) (**Figure 1E**). The gastric lumen was remarkably narrowed with slight blurring of the plasma membrane layer; Multiple intrahepatic masses demonstrated hypointense signal on T1WI (**Figure 1D**), the hyperintense signal on T2WI (**Figures 1A, G**) and ring-like enhancement on DEC-MRI without hepatospecific contrast agent (**Figure 1C**: Hepatic Arterial Phase; **Figure 1F**: Portal Venous Phase; **Figure 1I**: Delayed Phase), which the larger mass (5.7×5.3 cm) located in the lower right posterior lobe (**Figures 1D, G**).Many enlarged lymph nodes were found in the hepatogastric space and mesenteric zone. In contrast, no apparent lesion was discovered in the pelvic region (**Figure 1J**).

**Figure 2:**
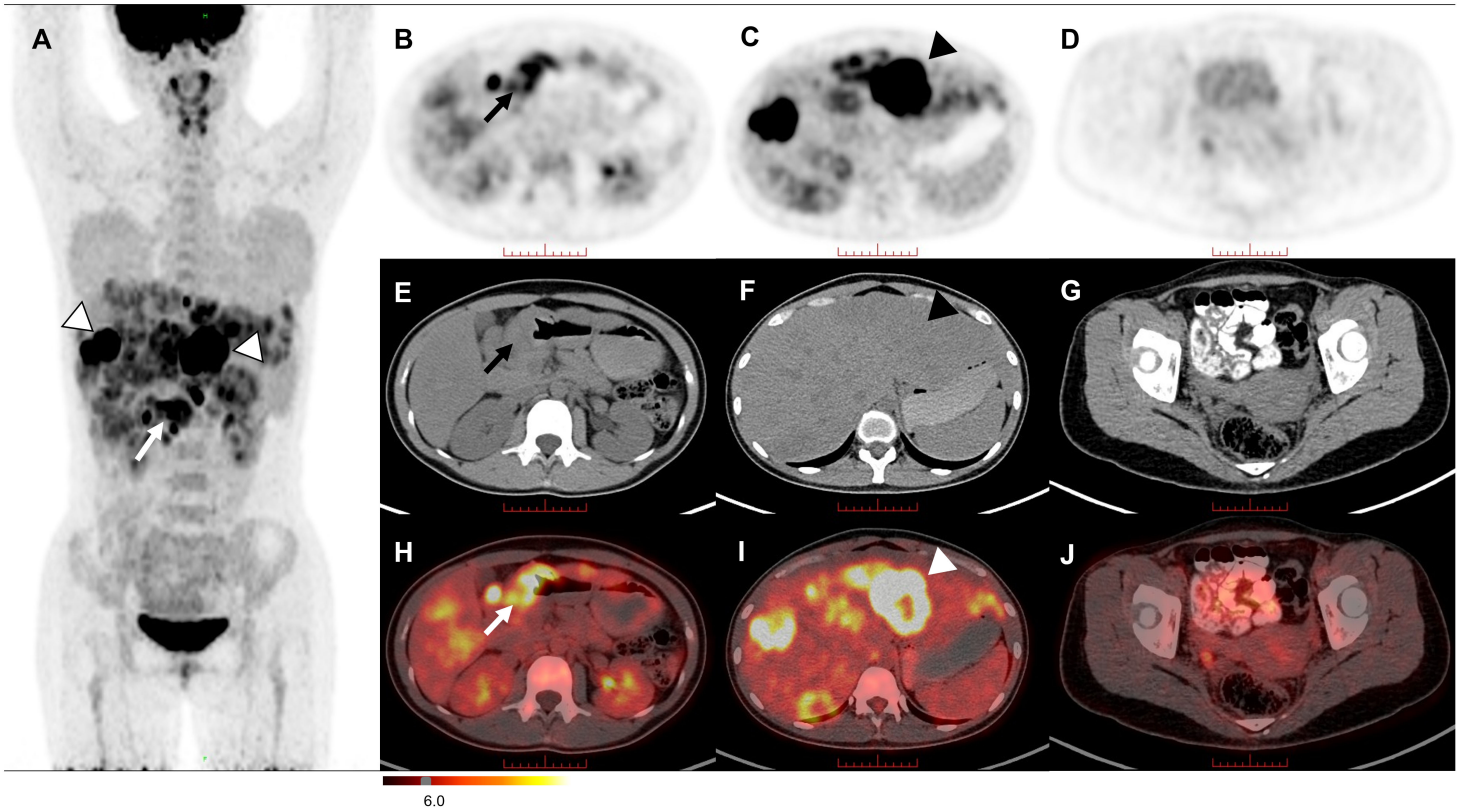
The MIP image **(A)** revealed intense uptake in the stomach (arrow) and liver (triangles). On axial views, remarkably ring-shaped high uptake was observed in the thickened gastric sinus wall [**(B, E, H)** arrow] and the left lobe of the liver [**(C, F, I)** triangle]. No significant FDG uptake was observed in the pelvic region **(D, G, J)**.

The ^18^F-fludeoxyglucose (^18^F-FDG) PET/CT revealed intense uptake in the stomach and liver, and moderate to intense uptake in the lymph nodes of the hepatogastric space and hilar region (**Figure 2A**). On axial views, remarkably ring-shaped high uptake was observed in the thickened gastric sinus wall (SUV_max_, 7.1; **Figures 2B, E, H**) and the left lobe of the liver (SUV_max_, 27.1; **Figures 2C, F, I**). No obvious abnormal uptake lesion was investigated in the pelvic region (**Figures 2D, G, J**). These imaging findings suggested a gastric malignancy with liver metastases, which in combination with the clinical history is considered to be PGC (T_4b_N_3a_M_1_, IV). Subsequently, pathology and immunohistochemical analysis supported this diagnosis, with immunohistochemical of hGH (–), AFP (-), SALL4 (+), Brg-1 (SMARCA4) (+), INI-1 (+), P40 (+), NUT (-), Glypican-3 (-), HepPar-1 (-), HCG (+), SOX2 (-), OCT-3/4 (-), CD30 (-), PLAP (+) and HSD3B1.

**Figure 3:**
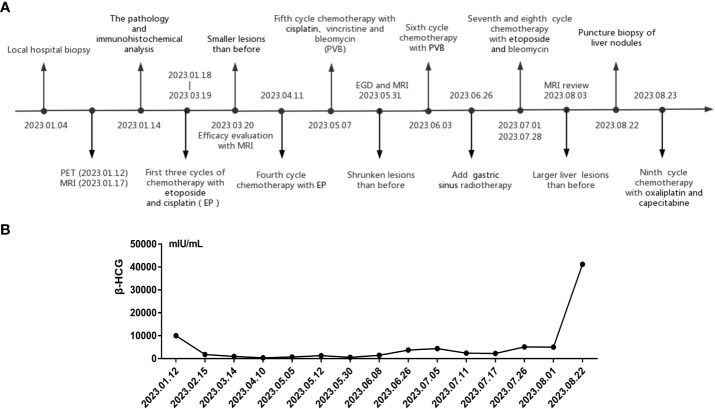
The treatment process timeline **(A)** and β-HCG value **(B)** within nine cycles of chemotherapy.

After the definitive diagnosis was established, the patient underwent three cycles of chemotherapy with etoposide and cisplatin (EP). Upon completion of the third cycle, an MRI and β-HCG were scheduled to assess treatment efficacy, which MRI demonstrated smaller lesions than before, and the β-HCG dropped to 983.2 mIU/ml after treatment (**Figure 3**). Further, in the fifth cycle of chemotherapy, we changed a new chemotherapy regimen (cisplatin, vincristine and bleomycin, PVB) to mitigate the risk of drug resistance. Before the sixth cycle treatment, a reassessment of the patient showed further shrunk lesions observed through esophagogastroduodenoscopy and MRI with β-HCG declined to 584.1 mIU/ml. However, following the completion of the sixth cycle (PVB), the β-HCG levels began to rise, reaching 1465.0 mIU/ml. Recognizing the potential for disease progression, additional radiotherapy (gastric sinus 45Gy/25f/36d, 1.8Gy/f) was introduced, alongside chemotherapy. For the seventh and eighth cycles, the chemotherapy regimen was adjusted to bleomycin and etoposide (EB) to prevent accumulation of cisplatin toxicity. Despite these interventions, β-HCG exhibited a sustained upward trend, peaking at 41170 mIU/ml in the final assessment (**Figure 3B**). MRI results indicated an enlargement of liver lesions and a reduction in gastric sinus lesion (**Supplementary Figures 1–3**). Following a multi-disciplinary approach, the ninth cycle of chemotherapy was modified to include oxaliplatin and capecitabine. Consequently, the patients underwent a liver nodule puncture biopsy, revealing the following results: Ckpan (AE1/AE3) (+), CK8/18 (+), P63 (+), HCG (part+), SALL4 (+), PLAP (+), hGH (-), HepPar-1 (-), Arginase-1 (-), Glypican-3(-) (**Supplementary Figure 4**). Throughout the treatment course, most tumor markers such as CEA, AFP, and CA125 remained within normal ranges, except for a persistent increase in CYERA21-1 and a decrease in NSE. Moreover, we summarized a table and figure to visualize the hematology parameter changes of pre-treatment and post-treatment during the treatment course (**Table 1**; **Supplementary Figure 5**). The results indicated a losing monocyte counts which may be related to the increased death of drug loaded macrophages depleting monocyte. To provide a clear overview of the treatment journey, we have summarized the entire process in a timeline (**Figure 3A**). In summary, the results suggest a prognosis similar to the majority of choriocarcinomas, indicating a poor outcome. Our team will continue to closely monitor this patient’s progress.

**Table 1:** The haematology parameter changes of pre-treatment and post-treatment during the treatment course.

cycle of therapy	WBC (10^9/L)		Neutrophil (10^9/L)		LYMPH (10^9/L)		MONO (10^9/L)		MONO% (%)		RBC (10^9/L)		HB (g/L)		PLT (10^9/L)	
	Pre	Post	Pre	Post	Pre	Post	Pre	Post	Pre	Post	Pre	Post	Pre	Post	Pre	Post
1	9.11	6.53	7.16	5.71	1.27	0.66	0.61	0.1	6.7	1.5	2.98	3.47	69	94	239	181
2	6.53	3.42	1.79	2.78	1.43	2.15	0.17	0.09	4.9	1.7	3.72	3.58	106	102	184	307
3	4.47	10.18	1.94	6.3	1.95	3.51	0.44	0.31	9.9	3	3.81	3.85	118	122	198	251
4	3.18	7.09	0.88	3.09	1.76	3.62	0.45	0.27	14.1	3.8	3.75	3.97	122	130	116	183
5	3.65	7.67	1.29	3.87	1.91	3.4	0.34	0.35	9.3	4.6	3.53	4	115	132	119	185
6	4.71	7.38	2.29	4.37	1.67	2.42	0.53	0.49	11.3	6.6	3.46	3.47	112	113	78	264
7	7.41	5.07	5.76	3.83	1.16	1.02	0.41	0.16	5.6	3.2	3.22	3.1	104	100	86	210
8	5.08	7.21	3.83	6.47	0.58	0.48	0.62	0.22	12.3	3.1	3.02	2.52	95	81	115	122

Pre, pre-treatment; Post, post-treatment; WBC, white blood cell count; Neutrophil, neutrophil count; Lymph, lymphocyte count; Mono, monocyte count; Mono%, percentage of monocyte; RBC, red blood cell count; HB, hemoglobin; PLT, platelet count.

## Discussion

PGC is a rare and highly malignant epithelial tumor of trophoblastic origin, primarily found in the gastric sinus, followed by the gastric body and cardia (7–10). The histogenesis and pathogenesis of PGC remain incompletely defined, with various speculations, most of which suggest that it is derived from the dedifferentiation of gastric adenocarcinoma cells (11). Clinical manifestations of PGC lack specificity and closely resemble those of gastric adenocarcinomas, including symptoms such as vomiting blood, black stools, anemia and epigastric pain (12). PGC is more likely to cause gastrointestinal bleeding than other tumors (13). In our case, the patient initially presented with no overt symptoms, but was diagnosed with severe anemia upon admission, with hemoglobin of 69 g/L. The β-HCG may serve as a specific tumor marker for choriocarcinoma, aiding in diagnosis and treatment evaluation (14). In this case, the β-HCG declined following the initial several courses of chemotherapy, but subsequently rose during follow-up treatment, a less favorable treatment response.

Similar to choriocarcinoma, PGC is a highly malignant tumor with a propensity for early hematogenous spread and an overall grim prognosis, with most patients surviving for only about six months (15, 16). The timely diagnosis of PGC is critical for potentially improving patient outcomes. However, diagnosing PGC remains a considerable challenge due to its low incidence and the absence of specific symptoms. The diagnostic process for PGC necessitates the exclusion of other occult primary lesions besides gastric tumors. In this context, ^18^F-FDG PET/CT, a noninvasive multifunctional imaging modality, offers distinct advantages compared to MRI. It excels in assessing the systemic involvement of patients by not only identifying the tumor metastasis site but also detecting occult primary lesions. On the other hand, MRI provides more precise insights into the relationship between lesions and the surrounding tissue. The typical PET imaging of primary choriocarcinoma (such as primary mediastinal choriocarcinoma) shows a ring-shaped high FDG uptake with a central radioactive deficit, which may be associated with the absence of interstitial vascularity and large central necrosis in the lesions (17). In our case, PET/CT imaging of the patient displayed a similar characteristic with intense ring-shaped uptake in both the liver and gastric sinus lesions. Thus, when a ring-shaped hypermetabolic gastric mass and liver mass are detected on PET/CT with a remarkably elevated HCG value but lacks history and signs of pregnancy-associated choriocarcinoma, the PGC should be considered as a differential diagnosis.

In conclusion, this study unveils a rare case of primary gastric choriocarcinoma with multiple hepatic metastases. ^18^F-FDG PET/CT has unique advantages in assessing the systemic involvement of patients, and ring-shaped high FDG uptake with central radioactive deficit may be the typical PET imaging of PGC.

## Data availability statement

The original contributions presented in the study are included in the article/**Supplementary Material**. Further inquiries can be directed to the corresponding author.

## Ethics statement

Ethical approval was not required for the study involving humans in accordance with the local legislation and institutional requirements. Written informed consent to participate in this study was not required from the participants or the participants’ legal guardians/next of kin in accordance with the national legislation and the institutional requirements. Written informed consent was obtained from the individual(s) for the publication of any potentially identifiable images or data included in this article.

## Author contributions

In this paper, YZ and WD conceived manuscript. YZ carried out manuscript preparation. ZC and WD revised the manuscript. SL and MY provided constructive suggestion. ZC guaranteed the integrity of the entire study.
