# Supplementary material for: Case Report: a rare primary gastric choriocarcinoma revealed on 18F-FDG PET/CT
Source: Front Oncol. 2023 Nov 9;13:1227236. doi: 10.3389/fonc.2023.1227236 (PMC10666916; doi:10.3389/fonc.2023.1227236)
Supplement: Supplementary file 1 [file DataSheet_1.docx]

Supplementary Material

- **Case report: A rare primary gastric choriocarcinoma revealed on ^18^F-FDG PET/CT**

Yi Zhao^1,2†^, Wei Diao^2†^, Suping Li^2^, Mengxi Yang^3^, Zhuzhong Cheng^1*^

*** Correspondence:** Zhuzhong Cheng: zhuzhongcheng@yeah.net

# Supplementary Figures

#
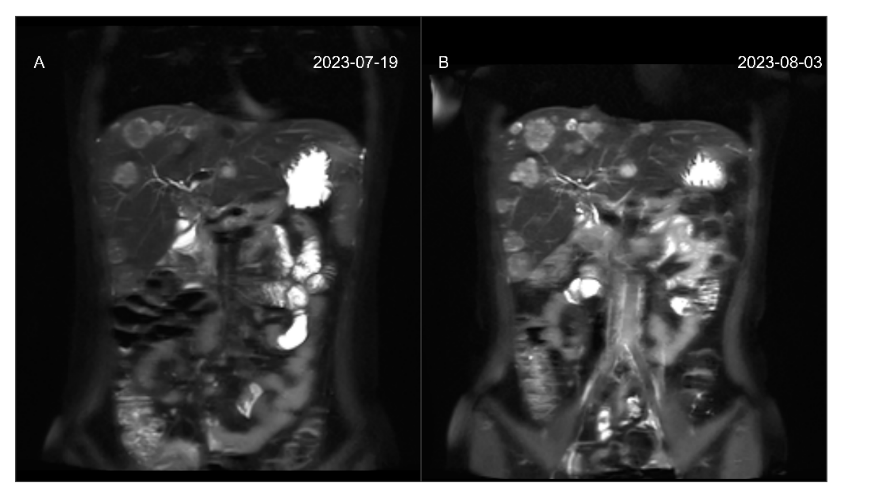


**Supplementary Figure 1.** The coronal view of comparison before (A) and after treatment (B) in MRI (T2WI), which indicated the increased and larger liver lesions.


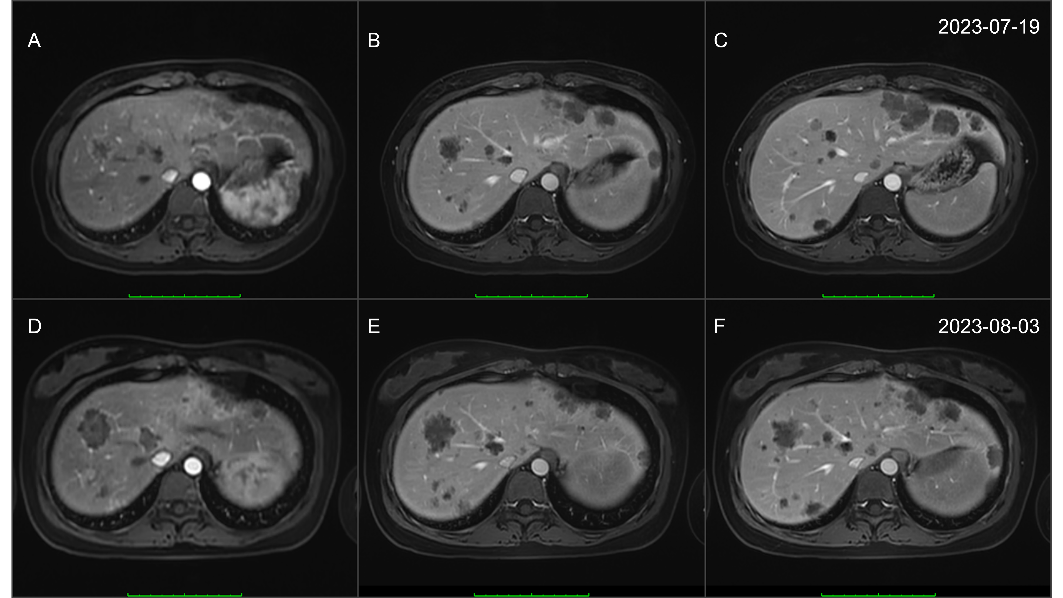


**Supplementary Figure 2.** The axial view of comparison before (A-C) and after (D-F) treatment in DEC-MRI (The first column: Hepatic Arterial Phase; The second column: Portal Venous Phase; The third column Delayed Phase)


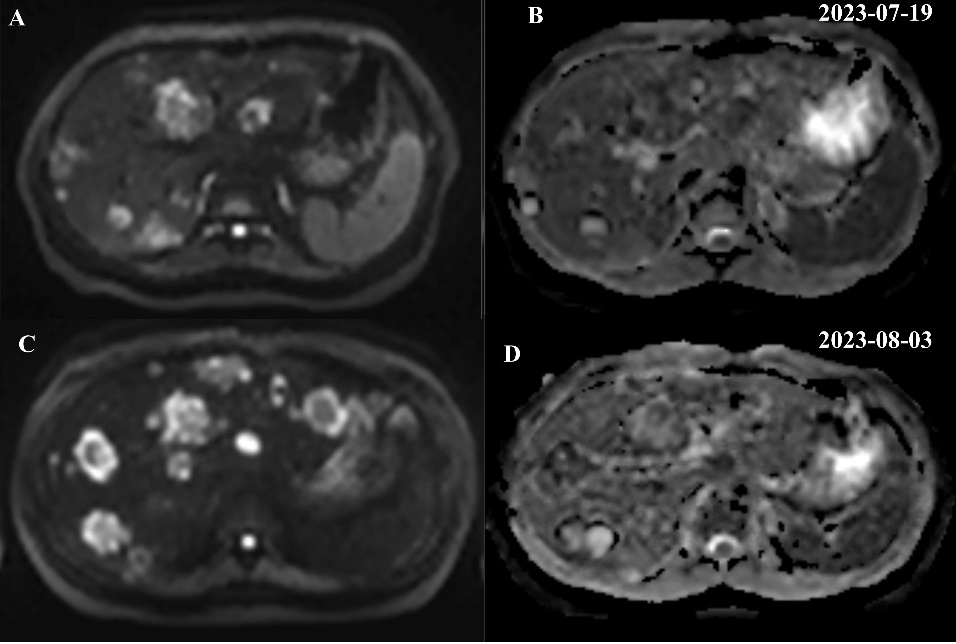


**Supplementary Figure 3.** The axial view of comparison before (A, B) and after (C, D) treatment in DWI (A, C) and ADC (B, D).


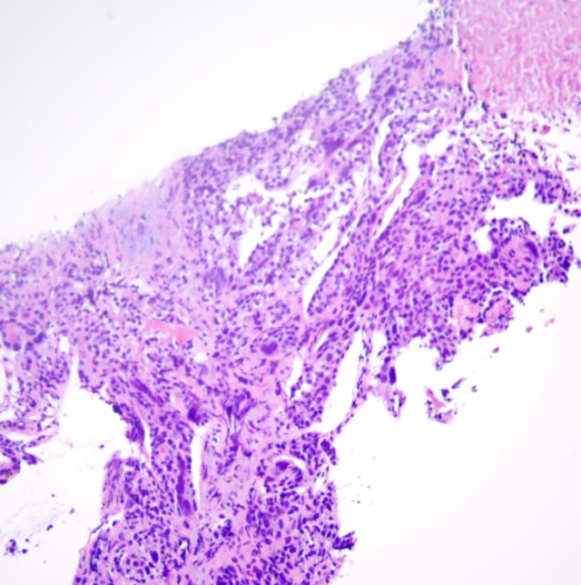

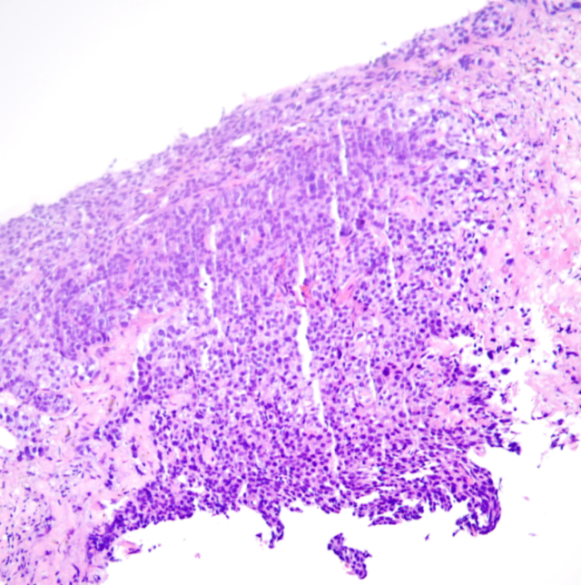


**Supplementary Figure 4.** The pathological pictures (HE stains) of liver nodules in the last time.


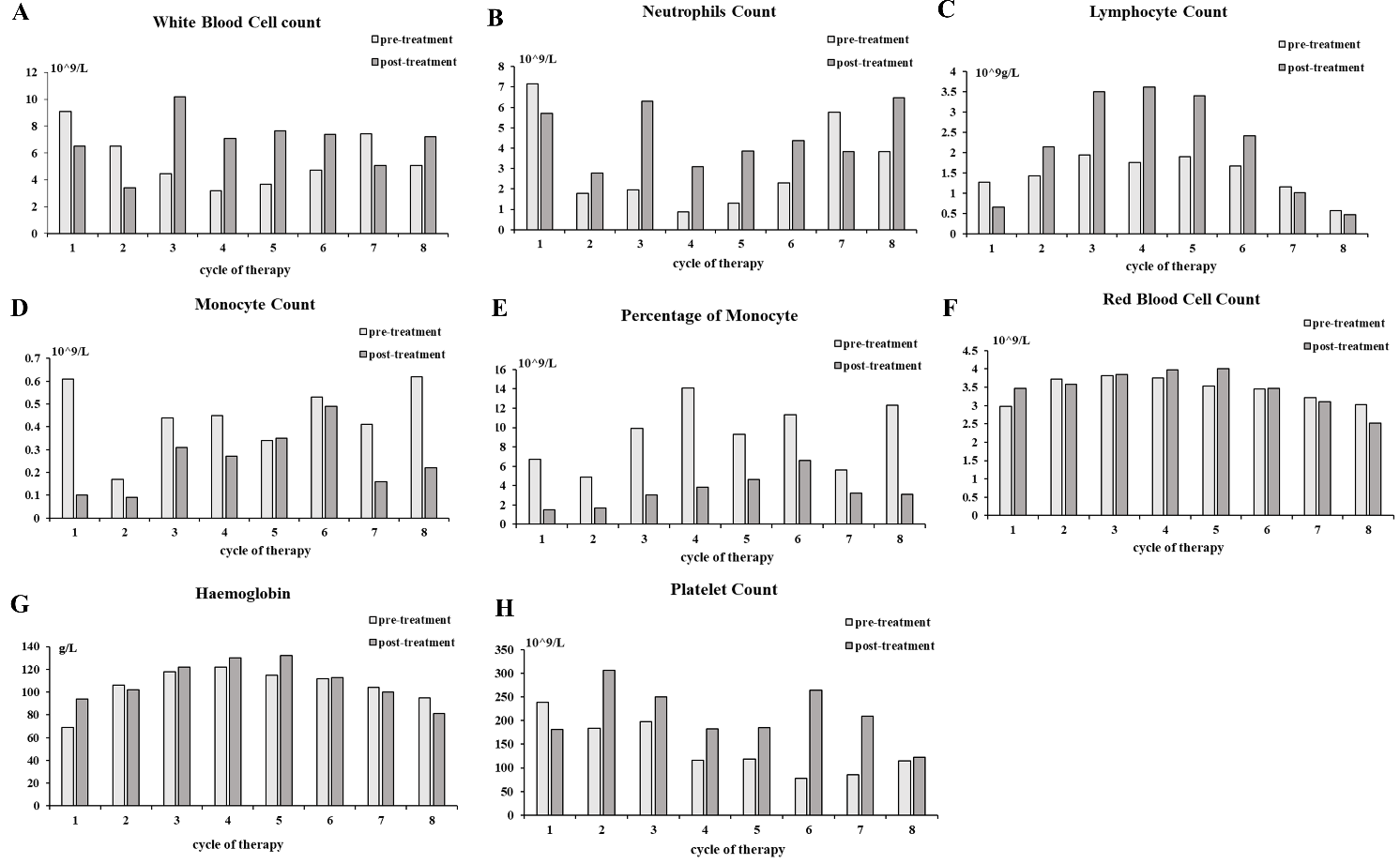


**Supplementary Figure 5.** The haematology parameter changes of pre-treatment and post-treatment during the treatment course
